# Supplementary material for: Differential chromatin accessibility landscape reveals structural and functional features of the allopolyploid wheat chromosomes
Source: Genome Biol. 2020 Jul 19;21:176. doi: 10.1186/s13059-020-02093-1 (PMC7368981; doi:10.1186/s13059-020-02093-1)
Supplement: Supplementary file 5 — Additional file 5. Review History. [file 13059_2020_2093_MOESM5_ESM.docx]

**Review history**

**First round of review**

**Reviewer 1**

The authors describe the differential accessibility of chromatin and relationship with wheat genomic features, functions and structure. This excellent paper not only provides new and important information about the wheat genome, but novel insights into how the structural organisation of a recent polyploid plant, with extensive repetitive DNA and transposable element content, influences gene expression in different sub-genomes and regions. The analyses are conducted thoroughly and there are many results; each adds a valuable and well-described insight.

I just have one concern which is with the sections of the paper dealing with the partitioning of genetic variance for phenotypic traits in regions with different chromatin accessibility (l. 39-40, l. 538-550, Fig. 10, Additional file 4, l. 667-679). Although the decile scores for chromatin accessibility were from the whole genome, the SNPs are based on exome sequencing. As the authors have shown, the accessibility of the gene coding portion of the genome differs and relationships are non-linear with distance from coding genes. I am not convinced that the genotype-phenotype matrix can be extrapolated genome-wide in that case, as there is little contribution from non-coding regions. I appreciate that the authors have been thorough in selection of sequences, states, randomizing and analysis, I'm just not sure that the hypothesis can be adequately tested with this dataset. For example, if a large proportion of phenotypic variance derived from differently accessible TEs (close and far from genes, potentially active/ regulatory) would it be found here? What would be the control? Fig. 10 and additional file 4 also show that the correlations between decile score for accessibility and variance explained are highly variable and the trend is not clear and convincing to me yet; e.g. for the HD trait in both years. The trend seems clearer where less variance is explained which makes me worry that a large number of 0 scores influences the results. At l. 549, the authors give the range and % increase for the bottom and top deciles, but an appropriate statistical test of the variation is needed and the limitation of using the exome-derived SNPs should be discussed. Alternatively, I think these sections could be deleted without damaging the paper and its already extensive and important conclusions.

A few, minor suggestions and a few typing mistakes I spotted are listed below. Otherwise I congratulate the authors on their research and for a well-written and fascinating paper, and have no other improvements to suggest.
Minor points:
Fig. 1c. The darker colour for chromosome group 4 DNS values is not very clear. Perhaps these could additionally be hatched, or another symbol used.
Fig.2. b is below, rather than to the right of, a, which is not logical with the rest of the panel order. Perhaps a 2 panel x 3 row figure would work better.
Fig. 2f and Fig. 8. As the red, blue, purple colour scheme has already been used for A, B and D genomes, different colours for these different entities would be less confusing.
Fig. 6. The panels should be aligned for the y-axes to make the differences easier to see.
l. 102, 445 and 597. Delete the phrase 'aimed at'
l. 448. Is 'formally' meant rather than 'formerly'?
l. 524 and l. 530. Correct 'conincided
l. 528. Correct 'charactristic'
l. 538. Correct 'differect'
l. 623. Add 'of' TE composition

**Reviewer 2**

The paper reports the analysis of differential MNase-seq data derived from the wheat genome.  They calculate differential nuclease sensitivity scores to interpret the data. To me the additional value of calculating the DNS scores for all the analyses the authors performed is not clear. A large part of the paper draws conclusions that are already reported by others (without calculating DNS scores). Calculating DNS scores in this manuscript did not reveal new insights for those analyses, and did therefore not result in a significant advance over previously published studies. The data are also sometimes also overinterpreted: for example, correlations are often reported as causal relationships.
The finding that the accessibility of TEs is related to the relative position of TEs on the chromosome (pg 17) could be an original result. The same is true for the conclusion in lines 498-499 (distance between genes affects DNS scores, but proximity to centromere seems also relevant).
It is a pity that a reader first has to go through a lot of elaborately explained analyses that recapitulate findings by others before getting into the analyses that do provide newer insight into the features playing a role in chromatin accessibility along the chromosome, such as distance between genes, type of TEs, and proximity to the centromere.  
I miss a discussion about the function of MSFs located distantly from the coding regions. The authors should also have identified putative regulatory regions by their MNase-seq approach. The authors however do not discuss this at all. They could for example compare their data with that of ref 14.

From page 10 on my feedback is less detailed.

Details
*     The text needs to be improved; there are sentences that are grammatically not correct and relatively often the content or formulation is incorrect. Below I provide examples in the introduction. Please use this to improve the rest of the text as well.
*     53: open and closed should refer to chromatin, not to nucleosomes. The use of accessible is in my view better than 'open'.
*     57. On top of transcriptionally inactive heterochromatin, inactive genes are also transcriptionally inactive (silenced by Polycomb silencing).
*     59-61. Not only TEs shape the 3D genome. (active) CREs interact with their target genes. The message in the sentence does not cover what is known from literature.
*     61-63. This is not what is said in reference 9.
*     64-67. This sentence is an overstatement. There is much more known about the role of chromatin in the regulation of gene expression, also based on other assays than accessibility assays. Think about ChIP-seq studying histone modifications or the binding of TFs, DNA methylation profiles, etc, etc. Also, accessibility alone is not sufficient to study the principles underlying nucleosome organisation. The references should also include ref 14.
*     81-87. This is a strange statement. Intergenic does not mean automatically transposons. There are numerous unique intergenic regions. At the same time there are indeed accessible sequences within transposable elements. There are more papers showing DHS sites in distant intergenic regions in crop plants. See e.g. Wang et al Nat Genetics 2017 (cotton); Oka et al 2017 Gen. Biology (maize). In animals there are numerous of such stories, so emerging is an understatement.
*     91-96. Sentence is not formulated correctly. Example is 'sensitivity to DNAse I treatment or transposon accessibility'? The latter is measured by e.g. sensitivity to DNAse I treatment, so why are these two different subjects in the sentence?
*     96-98 Here it is mentioned that no such dependence…wheat, while the sentence above suggests this is the case. So the two sentences seem to contrast each other. In addition in the first part of the sentence it is not mentioned this is referring to TE (DNA?) methylation while this is suggested in the second part of the sentence.
*     112-114. It is not clear to me why the message in the first part of the sentence would indicate that the gradient of TE abundance is the product of long-term selection.
*     124. Methylation can also be histone methylation better to add 'DNA'.
*     129-131. Table S1 does not show 1.2 billion reads. There are other numbers in S1. Would be good to indicate the average number per library. What is the size of the genomic intervals?
*     140-142. The higher level in the D genome is only slightly higher when looking at the separate type of sequences. By the way, why are those numbers at the bottom of the table when they are discussed 2nd in the second?
*     142-144. Where can we see the info on chromosomes-> Table S2. Mention that here already.
*     Table S2. How come most of the genome is not covered by MSFs & MRFs? What is the rest?? Please address this issue.
*     150-154. This needs more introduction. One needs to be able to read the paper without having to go to other papers to understand what is examined. How are the features as gene density, recombination etc distributed over the 5 different type of segments? Which segment type is enriched for TEs? What is the difference between distal and interstitial? What is the difference between R1 and R3? Between 2a and 2b? Do these 5 type of segments cover the entire genome?  A figure in the supplemental may be handy, also later in the text you use the same annotations and without a figure it may be hard to follow for non-wheat scientists.
*     154 Where does the proximal come from? Which segment type is 'proximal'?
*     156-157.Which 'other features'? Other relative to what? The conclusion drawn is not clear to me. Also here you calculate DNS scores, so based on what can you draw this conclusion? What the do results really mean?
*     Figure 1c: 0 is missing on Y-axis.
*     162-163. Where can I see this decline over the chromosome? Reference to a Table should be overhere. And how can you see the decline over the chromosome while MSFs & MRFs only cover a tiny part of the genome?
*     167. Table S3 does not show the information as stated in the text. Nothing about a gradient over the chromosome.
*     170-171. I don't see data supporting the conclusion. If the segments close to the centromeric region would be placed close to the end of the chromosomes, they may be as inaccessible as they are now. It is likely that the sequences within the segments for a large part determine the accessibility.
*     175-182. The text is difficult to follow without a figure.
*     181-193. The R1 region of chromosome 4A still shows a much lower DNS score than the average DNS score for R1 segments, and is in between R1 and R2a. In other words the sequence in this region matters for its DNS score, not (just) the position within the chromosome. After the translocations and inversions, I assume the sequence of the segment that now makes up R1 may also have been changed a bit. You also conclude yourself that this R1 segment is much less accessible. In other words your hypothesis in lines 170-171 does not hold.
*     194-205. What is the added value of calculating the DNS scores for the known data indicating that accessibility is positively correlated with recombination rates?
*     213: Table S4 should probably be S5.
*     FigS3-S9: the top and bottom panels are intermingled, so cannot be called top and bottom panels.
*     235-236. Large distances from genes does not automatically mean that regulatory sequences are overlapping with repetitive sequences. There are also unique sequences far from genes. One has to do the overlap with repetitive sequences to draw conclusions.
*     237-244. All kind of data are mentioned without a reference to a figure/table.
*     257-261. Open chromatin at TSSs and TESs is not epigenetic. It is dependent on the expression level of genes. It has nothing to do with heritable changes to the chromatin structure.
*      Figure 2 does not indicate the location of the transcription stop sites.
*     305. 19% increase relative to what?
*     316. Reduction relative to what? Region has even lower DNS values than region d.  
*     329. I do not see the data for the 500 bp upstream of the CDS in Figure 4.
*     338. A significant DNS score difference compared to what? among which regions?
*     384-385. Cause & effect relations are not shown. So one can only say there is a correlation, not that the position does affect the accessibility.
*     394-396. There seem to be more with a positive score. e.g. Sakura.
*     408-410. There is a third with positive scores DTC famc 26.
*     414. Which Gypsy TEs are overrepresented in gene promoters?
*     413-423. I miss references to tables/figure.
*     422-423. You cannot say anything about what is causal. You can only conclude that the average proximity of TEs to genes seems to be positively correlated with their accessibility. This is also true for 441-446. There is a clear correlation, but you do not proof that TEs are the cause of decreased accessiblity of the CDS. Furthermore, this is also true for messages lateron (e.g. 449-450).
*     490-498. The text suggests that after showing data in 8e another analysis is done, which you would then expect to end up in another figure panel, but again 8e is being referred to. This is confusing. Then another comparison is done referring to 8f, whereby it is unclear what is different with the previous comparison (different intergenic intervals from distal and centromeric regions).
*     498-500. Are TEs with lower DNS scores more concentrated close to the centromere? Does this explain the observed lower DNS score the closer to the centromere (even for genes with a distance of <100 kb)?
*     500-504. It is unclear to me how the data on chromosome 4A can support the finding stated in 498-500. The average distance between genes is very similar, while R1 of 4A is distal, like R1 of 4B and 4D. Still R1 of 4A has lower DNS scores. Maybe R1 of 4A has more TEs that result in lower DNS scores?
*     536-537. I do not see any CENH3 mapped reads in Fig 9.
*     Fig 10. Which deciles represent low vs high DNS scores? Where do the SNPs come in? The deciles are for the DNS scores.
*     571. What do you mean with higher TE activity? That they amplify more (enlarging the genome)? Something else?
*     572. Where do you show the D genome has an increased gene density? On page 20 it is stated that the A genome has an 8 fold increase in gene density at distal regions while the D genome has a 6 fold increase in gene density.
*     579. Non-nucleosomal footprint sounds vague.
*     580. It would be best to add 'proximal' to upstream regulatory regions. 2 kb is still very closeby.What about MSFs further upstream than 2 kb?
*     587. Bias in gene expression levels?
*     586-589. This is not just true for wheat, also for other organisms, including animals.
*     590-591. You observed a correlation.
*     598. The type of TEs is not addressed in the sentences before. So better use 'our' results in 596.
*     616-619. Your data in figure 1c indicated there is some change towards more open chromatin.
*     633. TE silencing is most effective when TEs are retained in the genome: this sentence is confusing. Compared to what?  IS there another option than being in the genome?
*     634-638. The messages here are also not entirely clear. For example what is meant with removal? Removal through which process? Recombination? A cut mechanism without being followed by the 'paste' part of the process? why would recent TE bursts be accompanied by less effective removal of TEs from low recombining regions?
*     642-643: also true for ref 49, 51 (and others)
*     652-655. Figure 1 and 9 indicates the highest scores are observed at the chromosome ends.
*     663-666. Can you explain why the DNS scores and Cereba LTR density would be affected by an artifact of genome assembly and the immunoprecipiation signal not?
*     676. See als Ricci..Schmitz..Zhang et al and Lu … schmitz et al 2019 (Nature plants).
*     679. In Oka et al DNase I-seq data predict enhancers genome wide, not only for the genes indicated.

**Authors’ response to reviewers**

Comments from Reviewer #1:

1. Comment 1: I just have one concern which is with the sections of the paper dealing with the partitioning of genetic variance for phenotypic traits in regions with different chromatin accessibility (l. 39-40, l. 538-550, Fig. 10, Additional file 4, l. 667-679). Although the decile scores for chromatin accessibility were from the whole genome, the SNPs are based on exome sequencing. As the authors have shown, the accessibility of the gene coding portion of the genome differs and relationships are non-linear with distance from coding genes. I am not convinced that the genotype-phenotype matrix can be extrapolated genome-wide in that case, as there is little contribution from non-coding regions. I appreciate that the authors have been thorough in selection of sequences, states, randomizing and analysis, I'm just not sure that the hypothesis can be adequately tested with this dataset. For example, if a large proportion of phenotypic variance derived from differently accessible TEs (close and far from genes, potentially active/ regulatory) would it be found here? What would be the control? Fig. 10 and additional file 4 also show that the correlations between decile score for accessibility and variance explained are highly variable and the trend is not clear and convincing to me yet; e.g. for the HD trait in both years. The trend seems clearer where less variance is explained which makes me worry that a large number of 0 scores influences the results. At l. 549, the authors give the range and % increase for the bottom and top deciles, but an appropriate statistical test of the variation is needed and the limitation of using the exome-derived SNPs should be discussed. Alternatively, I think these sections could be deleted without damaging the paper and its already extensive and important conclusions.

Response: We agree with the reviewer that we might have biased SNP dataset that likely underestimates phenotypic contribution of non-coding variants. Therefore, we have focused our analyses on the estimation of SNP effects only from the genic regions (gene body plus 1 kb flanking regions) showing different levels of chromatin accessibility. This analysis does not allow for discriminating the effects of variants in the intergenic and genic regions, but does allow us to assess the effect of variants located in the genic regions with different levels of chromatin accessibility. The respective sections in the Results, Methods and Discussion sections have been revised to reflect this.

We reran the analysis using only SNPs that are located within the gene body or within 1kb flanking regions. These SNPs were placed into 5 bins based on DNS region they fall in as ranked from most closed to most open 10 bp segments. The 5 bins represent the most closed regions (0-20%, 20-40%, 40-60%, 60-80%, and most open >80%), we chose to focus analysis on only the most open and closed regions. To take into account that more SNPs land in regions that more open than closed, we randomly selected 10,000 SNPs from each group and calculated the variance of phenotypes based on the included SNPs for these 2 regions. We statistically tested the distributions using pairwise Wilcoxon test to calculate the differences in proportion of variance explained in the 2 groups. For all comparisons p-values < 10-6. Overall, we find more than a 3-fold difference in the proportion of variance explained across the 10 traits measured.

Minor comments

2. Comment 2: Fig. 1c. The darker colour for chromosome group 4 DNS values is not very clear. Perhaps these could additionally be hatched, or another symbol used.
Response: Corrected. We lightened the colors for group 4 in the new figure.

3. Comment 2: Fig.2. b is below, rather than to the right of, a, which is not logical with the rest of the panel order. Perhaps a 2 panel x 3 row figure would work better.
Response: Due to comments from reviewer 2, we removed this figure from main text, and only refer to the correlations in the text. In light of bringing novel findings forward, we focused figure 2 to include a description of MSF and MRF regions found in our dataset.

4. Comment 2: Fig. 2f and Fig. 8. As the red, blue, purple colour scheme has already been used for A, B and D genomes, different colours for these different entities would be less confusing.
Response: Figure 2f is no longer included in the main figures. We changed the colors in figure 8, to little blue, apricot, and teal.

5. Comment 2: Fig. 6. The panels should be aligned for the y-axes to make the differences easier to see.
Response: Corrected.

6. Comment 2: l. 102, 445 and 597. Delete the phrase 'aimed at'
Response: Corrected.

7. Comment 2: l. 448. Is 'formally' meant rather than 'formerly'?
Response: Corrected.

8. Comment 2: l. 524 and l. 530. Correct 'conincided
Response: Corrected.

9. Comment 2: l. 528. Correct 'charactristic'
Response: Corrected.

10. Comment 2: l. 538. Correct 'differect'
Response: Corrected.

11. Comment 2: l. 623. Add 'of' TE composition
Response: Corrected.


Comments from Reviewer #2:

We would like to thank this reviewer for the very thorough evaluation on nearly every aspect of the manuscript!!! These comments were extremely helpful for improving the manuscript.

1. Comment 1: The paper reports the analysis of differential MNase-seq data derived from the wheat genome. They calculate differential nuclease sensitivity scores to interpret the data. To me the additional value of calculating the DNS scores for all the analyses the authors performed is not clear. A large part of the paper draws conclusions that are already reported by others (without calculating DNS scores).

Response: The main focus of our study was to investigate the chromosome-specific patterns of chromatin accessibility and identify genomic features whose distribution is linked with the detected patterns. The patterns we observed can be described as follows: there is a gradient of overall chromatin accessibility along the telomere-centromere axis (Figs. 1b, 1c), and this gradient is mirrored only by the chromatin in the intergenic regions, while chromatin in the genic regions shows similar average chromatin accessibility measures along the chromosomes. To our knowledge, a thorough comparison of these patterns along the chromosomes and more or less systematic attempts to find genomic correlates of these patterns were not reported for other species. We considered these aspects of our analyses as potentially novel (see comment 3 by the reviewer).

However, before going into the detailed analyses of these chromosomal trends, we felt that a more in-depth description of chromatin accessibility patterns and other genomic correlates across the genic and intergenic regions, and at the boundary between TEs and genes is needed. These patterns are then described in the context of broader chromosome-level trends and were used to identify putative factors that might contribute to the observed chromosome-level chromatin accessibility patterns. While this manuscript structure creates the feeling that many reported trends are similar to those reported before, and that novel findings are hidden at the end of the manuscripts, it fulfills two purposes:

First, it allows us to describe this novel genomic data (to our knowledge, no differential MNase-seq analyses have been reported for wheat so far) at greater depth to make it more useful as a community resource. Consistency with the previously reported patterns indicates that DNS adequately reflects chromatin accessibility in the wheat genome and allows for comparing our detected DNS patterns with those reported using other chromatin accessibility analyses methods. Second, it allows us to connect DNS data with the previously detected trends and link them with the genomic correlates of chromosome-level chromatin accessibility gradients described in our study.

We tried to bring more focus into the manuscript by removing or shortening some sections. The section describing the relationship between recombination and DNS scores was removed from main text. The section describing the relationship between gene expression and chromatin accessibility was substantially shortened and a figure describing this data was completely removed. The abstract was revised to bring more attention to the novel findings reported in the manuscript (relationships between the relative chromosomal position and TEs chromatin accessibility, connection of chromatin accessibility along the chromosomes with distance between genes, TE type, TE composition, and proximity to centromere).

In addition, in the revised version, we have expanded our analyses to include the published epigenetics data (15 chromatin states identified by Li et al., 2019 Genome Biology) and focus on the genomic features that could be uniquely detected only by the DNS analyses. For example, this recent paper only classified 2.7 Gb of the entire wheat genome into the 15 chromatin states, we scored the entire 16 Mb with a DNS score, and classified the entire genome into MSF/MRF outliers. We found that the majority of MSF (56%) did not overlap with any of the 15 chromatin states, and also showed relatively small overlap with the chromatin states characterized by Li et al. 2019. In total, these unique MSF covered 99.5 Mbp, with 18,709 MSF (~1.6 Mb) located within the 2 kb promoter regions of 12,788 high-confidence gene models. These new analyses are presented in Fig. 2, and further discussed in the Discussion section.

During the discussion, and across the Results section, we have added references to two insightful papers describing the distal cis-regulatory elements in maize (Ricci et al., 2020, Nat. Plants; Lu et al., 2020, Nat. Plants). We should note, however, that these two studies were published 4 months after the submission of our manuscript to Genome Biology.

More specific details of revisions made could be found below, in the responses to specific comments.

2. Comment 2: The data are also sometimes also overinterpreted: for example, correlations are often reported as causal relationships.

Response: Thank for bringing this to our attention. This issue is probably one of the most common mistakes found in many descriptive genomics publications that do not use experimental manipulation with the system or carefully selected samples to establish causal links between genomic patterns and other biological attributes of a system. We revised the manuscript to remove this bias in data interpretation.


3. Comment 3: The finding that the accessibility of TEs is related to the relative position of TEs on the chromosome (pg 17) could be an original result. The same is true for the conclusion in lines 498-499 (distance between genes affects DNS scores, but proximity to centromere seems also relevant). It is a pity that a reader first has to go through a lot of elaborately explained analyses that recapitulate findings by others before getting into the analyses that do provide newer insight into the features playing a role in chromatin accessibility along the chromosome, such as distance between genes, type of TEs, and proximity to the centromere.

Response: (also see response to comment 1) This, as was mentioned in the response to comment 1, is partly caused by our attempt to describe this resource for community use, and putting results reporting more novel insights to the end of the manuscript. We have condensed these parts of the manuscript. Also, we have compared our DNS patterns with the published results (from Ref. 14) to emphasize features that could be detected using only DNS profiling (see Fig. 2).

From response to comment 1: “We tried to focus the manuscript by removing or shortening some sections. The section describing the relationship between recombination and DNS scores was removed. The section describing the relationship between gene expression and chromatin accessibility was substantially shortened and a figure describing this data was removed. The abstract was revised to focus attention to the novel findings reported in the manuscript (relationships between relative chromosomal position and TEs chromatin accessibility, connection of chromatin accessibility along the chromosomes with distance between genes, TE type, TE composition, and proximity to centromere). “


4. Comment 4: I miss a discussion about the function of MSFs located distantly from the coding regions. The authors should also have identified putative regulatory regions by their MNase-seq approach. The authors however do not discuss this at all. They could for example compare their data with that of ref 14.

Response: We have expanded this section to include the analyses of distant putative regulatory regions and their sequence composition and annotation. We also added a supplementary table the describes the 2.7Gb of the genome described in ref 14, and how those regions overlap with the entire genome DNS score, and MSF/MRF regions detected using DNS-seq technique.


Details
5. Comment 5. * 53: open and closed should refer to chromatin, not to nucleosomes. The use of accessible is in my view better than 'open'.
Response: Corrected here and throughout the manuscript.

6. Comment 6. *57. On top of transcriptionally inactive heterochromatin, inactive genes are also transcriptionally inactive (silenced by Polycomb silencing).
Response: We have added a reference to Polycomb-mediated silencing of gene expression.

7. Comment 7. 59-61. Not only TEs shape the 3D genome. (active) CREs interact with their target genes. The message in the sentence does not cover what is known from literature.
Response: Thank you for bringing this to our attention. We have added references, including some from studies done in maize that were published after our manuscript was submitted to Genome Biology.

8. Comment 8. 61-63. This is not what is said in reference 9.
Response: The reference was replaced with a more relevant one.

9. Comment 9. 64-67. This sentence is an overstatement. There is much more known about the role of chromatin in the regulation of gene expression, also based on other assays than accessibility assays. Think about ChIP-seq studying histone modifications or the binding of TFs, DNA methylation profiles, etc, etc. Also, accessibility alone is not sufficient to study the principles underlying nucleosome organisation. The references should also include ref 14.
Response: Absolutely agree with the reviewer that chromatin accessibility alone is not sufficient. In this sentence, we wanted to make the point that chromatin accessibility played an important role in epigenomic studies. We amended this sentence to indicate that other methods are also used, and modified sentence to make it more clear that that chromatin accessibility profiling, among other methods, was another important tool for studying chromatin. The reference to the wheat epigenome paper (former Ref. 14) is included.

10. Comment 10. 81-87. This is a strange statement. Intergenic does not mean automatically transposons. There are numerous unique intergenic regions. At the same time there are indeed accessible sequences within transposable elements. There are more papers showing DHS sites in distant intergenic regions in crop plants. See e.g. Wang et al Nat Genetics 2017 (cotton); Oka et al 2017 Gen. Biology (maize). In animals there are numerous of such stories, so emerging is an understatement.
Response: The section is revised to incorporate results from cotton (Wang et al Nat Genetics 2017) and maize (Oka et al 2017 Gen. Biology) studies.

11. Comment 11. 91-96. Sentence is not formulated correctly. Example is 'sensitivity to DNAse I treatment or transposon accessibility'? The latter is measured by e.g. sensitivity to DNAse I treatment, so why are these two different subjects in the sentence?
Response: We apologize for mistake. We implied sensitivity to treatment by transposase Tn5. The sentence has been revised.

12. Comment 12. 96-98 Here it is mentioned that no such dependence…wheat, while the sentence above suggests this is the case. So the two sentences seem to contrast each other. In addition in the first part of the sentence it is not mentioned this is referring to TE (DNA?) methylation while this is suggested in the second part of the sentence.
Response: There is no contradiction between the two sentences. In the first sentence, we point at the link between methylation (and other epigenomic phenomena) in the proximal cis-regulatory regions and expression bias between duplicated homoeologs. In the second sentence, we bring data from monkeyflower, where it was shown that such a bias in expression between homoeologs is associated with methylation transposable elements. No such connection between TE methylation and expression bias was observed in wheat.

13. Comment 13. 112-114. It is not clear to me why the message in the first part of the sentence would indicate that the gradient of TE abundance is the product of long-term selection.
Response: We apologize if the logic of this statement was not evident from what we have written. The estimate of divergence time among the three wheat genomes, A, B and D is about 5.5 million years, yet they have retained nearly complete collinearity in gene order. In contrast, the TE content in the regions between the syntenic genes has completely changed. This may only happen through a quite fast process of TE deletion and insertion that leads to complete replacement of intergenic TEs within short evolutionary timeframe. Comparison of the A, B and D genomes also showed that in spite of nearly complete replacement of TE space, the distance between the syntenic genes remained the same (PMID: 30115100). Considering fast TE deletion/insertion rate, these fixed distances should be under selection. Otherwise (intergenic distances are not under selection), we would expect quite a broad range of variation in intergenic distance lengths between collinear genes, due to stochastic loss or insertion of TEs, which can go in either direction, towards an increase or a decrease.

Part of this discussion comes from the previously published study on TE evolution in the wheat genome (PMID: 30115100). We have modified this section to add more clarity.

14. Comment 14. 124. Methylation can also be histone methylation better to add 'DNA'.
Response: The sentence is corrected as suggested.

15. Comment 15. 129-131. Table S1 does not show 1.2 billion reads. There are other numbers in S1. Would be good to indicate the average number per library. What is the size of the genomic intervals?
Response: In total, we produced 1.75 billion paired-end (PE) reads (= 3.5 billion reads), of which 2.4 billion reads were uniquely mapped to the genome (68% of total reads), which would correspond to ~1.2 billion PE reads. We added a new row in Table S1 to reflect average numbers for all stats generated. Genomic intervals for determining DNS score are 10 bp. The manuscript text was modified as follows: “…MNase digests were sequenced producing nearly 1.75 billion paired end (PE) reads (Table S1 in Additional File 1), of which about 1.2 billion PE reads were uniquely mapped to the wheat genome [35]. This dataset was used to calculate the differential nuclease sensitivity (DNS) scores for 10-bp intervals across the genome…”

16. Comment 16. 140-142. The higher level in the D genome is only slightly higher when looking at the separate type of sequences. By the way, why are those numbers at the bottom of the table when they are discussed 2nd in the second?
Response: This is an astute observation by the reviewer, and not reflected by the numbers in the Table. We find that intergenomic differences of DNS scores stems from the intergenic space, which we mention later in the text. Corrected, by removing sentence: “The higher overall chromatin accessibility level in the D genome compared to other genomes was also evident in the genic and intergenic regions (Table 1).”

17. Comment 17. 142-144. Where can we see the info on chromosomes-> Table S2. Mention that here already.
Response: Corrected.


18. Comment 18. Table S2. How come most of the genome is not covered by MSFs & MRFs? What is the rest?? Please address this issue.
Response: The details of how MSF and MRF were defined and described in the Materials and Methods section of the original manuscript. The program and statistical parameters that we used to discretely designate MSF and MRF was iSeg at a biological cutoff of sd=1.5 (one of the default cutoffs). This cutoff means that magnitude of the segment must be greater/less than 1.5 standard deviations of the genome wide distribution of data to be called significant (Girimurugan, et al, BMC Bioinformatics, 2018). In addition, using this biological cutoff, we ran both reps separately through the program, and filtered to include only regions that were considered outliers in both reps. Overall, we found that 1.26% and 1.53% of the wheat genome are considered MSF or MRF outlier regions (more than 1.5 standard deviations from the genome distribution). This means that the rest of the genome ~97% have DNS scores fall within the 1.5 deviation range and are unable to segmented from the rest of the genome. The maize paper (Rogers Melnick et al, PNAS, 2016), which used the same DNS score to designate hypersensitive and hyper resistant portions of the genome only reported 0.6% (19.4 Mb) of the maize genome as hyper-sensitive regions.

To avoid confusion, we have added some wording into the Results section to better define MSF and MRF. “Segmentation of the wheat genome was performed using the iSeg program [40] that, based on the distribution of DNS scores, can identify outlier regions (>1.5 standard deviations of the genome wide DNS score) corresponding to either MNase hyper-sensitive footprints (MSF) or MNase hyper-resistant footprints (MRF).”


19. Comment 19. 150-154. This needs more introduction. One needs to be able to read the paper without having to go to other papers to understand what is examined. How are the features as gene density, recombination etc distributed over the 5 different type of segments? Which segment type is enriched for TEs? What is the difference between distal and interstitial? What is the difference between R1 and R3? Between 2a and 2b? Do these 5 type of segments cover the entire genome? A figure in the supplemental may be handy, also later in the text you use the same annotations and without a figure it may be hard to follow for non-wheat scientists.
Response: We have added a panel into Fig. 1 that shows the trends in the distribution of various genomic features across five chromosome segments. Distal represents the ends, and interstitial are the middle 2 regions separating ends from pericentromeric region. Previous analyses (Choulet, et al Science, 2014) on chromosome 3B found that recombination rate is much higher on the distal ends, reduced in interstitial regions and nearly non-existent in the pericentromeric regions, gene density follows the same gradient, expression breadth, and TE composition follow the opposite gradient, with higher values in the pericentromeric region and reduced in the distal regions. Recent analyses (The International Wheat Genome Sequencing Consortium (IWGSC) 2018) showed all chromosomes followed the same trends for these features.

The amended text hopefully provides more clarity: “Previously, based on the distinct patterns of recombination rate, gene density, and gene expression distribution, each of the 21 wheat chromosomes was partitioned into five regions, referred to as R1 and R3 for the distal ends of the short and long chromosomal arms, respectively, R2a and R2b for the interstitial regions on the short and long arms, respectively, and the C region, which represents the pericentromeric region [35,37] (Fig. 1b). While R1 and R3 regions had high gene density and recombination rate, and reduced TE density, alternative splicing and gene expression variation, the R2a, C, and R2b regions showed opposite trends [34,35,37].“

20. Comment 20. 154 Where does the proximal come from? Which segment type is 'proximal'?
Response: In genetics literature, including model systems (e.g. Drosophila), chromosomal regions distal or proximal to centromere are often simply referred to as “distal” or “proximal”, omitting to mention every time that these locations are relative to centromere (“distal to centromere” or “proximal to centromere”). To reduce ambiguity, we replaced ‘proximal’ to ‘pericentromeric’ across the text.

21. Comment 21. 156-157.Which 'other features'? Other relative to what? The conclusion drawn is not clear to me. Also here you calculate DNS scores, so based on what can you draw this conclusion? What the do results really mean?
Response: These other features are recombination rate, gene density, TE composition, mentioned above. The amended text to hopefully provide more clarity is: “Overall, the pericentromeric and interstitial chromosomal regions each have negative DNS scores (Table 1, Fig 1b), while both distal regions possess positive DNS scores of 0.046 and 0.053 (i.e. open), mirroring the previously documented segmentation gradient for recombination rate, gene density, and gene expression, and a directly opposite gradient for TE composition.”

22. Comment 22. Figure 1c: 0 is missing on Y-axis.
Response: Corrected.

23. Comment 23. 162-163. Where can I see this decline over the chromosome? Reference to a Table should be over here. And how can you see the decline over the chromosome while MSFs & MRFs only cover a tiny part of the genome?
Response: Corrected by adding Fig. 1c, Table S3 reference to text. We apologize if this was not clear in the original version of the manuscript. This statement has nothing to do with MSF and MRF that are indeed discreet regions of the genome that have extremely high or low DNS scores (in other words these are outlier regions representing only small portion of the genome) identified by taking regions showing DNS scores 1.5 standard deviations away from the genome-wide mean. This has been discussed in response to Comment 18. This particular analysis was done using 2Mb window DNS scores, which show a clear gradient across the distal and pericentromeric segments. We clarified this in the text: “Overall, we observed a decline in chromatin accessibility along the centromere-telomere axis based on 2Mb window DNS scores spanning all chromosomes of all three wheat genomes (Fig. 1c, Figs. S3-10, Table S3).”

24. Comment 24. 167. Table S3 does not show the information as stated in the text. Nothing about a gradient over the chromosome.
Response: We admit using improper term (“gradient”) to describe data. The gradient of chromatin accessibility can be seen on Fig.1e. However, in Table S3, data was summarized using average DNS score for 5 chromosomal segments defined in Fig 1b. In fact, we just wanted to point at the differences in chromatin accessibility between the pericentromeric and distal regions of the wheat chromosomes. The text was modified by removing term “gradient”.

25. Comment 25. 170-171. I don't see data supporting the conclusion. If the segments close to the centromeric region would be placed close to the end of the chromosomes, they may be as inaccessible as they are now. It is likely that the sequences within the segments for a large part determine the accessibility.
Response: Indeed, sequence composition looks like to play role too, a conclusion reached later in the manuscript (though we still cannot rule out the impact of chromosome position). However, here and in the following paragraph we focused on exploring the impact of chromosomal position on accessibility using unique structural re-arrangement specific to only one of homoeologous chromosomes in group 4 (chr. 4A). We investigated the impact of sequence composition (TE types) and position in the next sections of the manuscript. We fixed the sentence indicating that chromosome position can be one of the possible factors affecting chromatin accessibility: “One of the likely factors affecting these differences in chromatin state between distal and pericentromeric regions is the position of a genomic region with respect to the centromere-telomere axis.”

26. Comment 26. 175-182. The text is difficult to follow without a figure.
Response: We have modified Fig. 1c to show structural re-arrangements on chromosome 4A relative to ancestral state still preserved on chromosomes 4B and 4D. Text was slightly modified for clarity.

27. Comment 27. 181-193. The R1 region of chromosome 4A still shows a much lower DNS score than the average DNS score for R1 segments, and is in between R1 and R2a. In other words, the sequence in this region matters for its DNS score, not (just) the position within the chromosome. After the translocations and inversions, I assume the sequence of the segment that now makes up R1 may also have been changed a bit. You also conclude yourself that this R1 segment is much less accessible. In other words your hypothesis in lines 170-171 does not hold.
Response: We should admit the sentence in lines 170-171 was not formulated properly, and represented one of the intermediate hypotheses. In the revised manuscript this sentence is modified (see response to Comment 25). But essentially, this is the conclusion (that by itself change of chromosome position does not immediately change chromatin state, even though position effect could not be ruled out entirely) that we have already outlined in the Discussion section of the original manuscript. We compared the TE sequence composition in R1 segment of chr. 4A with the R1 and R2a segments with other A genome chromosomes, and found that 4A-R1 is very similar to R2a (expectedly) and distinct from R1. This data indicate that sequence composition does have major effect on chromatin accessibility. We could not rule out effect of chromosome position in later analyses of the TEs that showed that the same classes of TEs show different accessibility depending on their position on chromosome.

28. Comment 28. 194-205. What is the added value of calculating the DNS scores for the known data indicating that accessibility is positively correlated with recombination rates?
Response: This section is removed in the revised manuscript.

29. Comment 29. 213: Table S4 should probably be S5.
Response: Corrected.

30. Comment 30. FigS3-S9: the top and bottom panels are intermingled, so cannot be called top and bottom panels.
Response: Thank you for noticing this mistake. Figure legends are modified.

31. Comment 31. 235-236. Large distances from genes does not automatically mean that regulatory sequences are overlapping with repetitive sequences. There are also unique sequences far from genes. One has to do the overlap with repetitive sequences to draw conclusions.
Response: Changed this part of the sentence to: ” … a considerable proportion of gene regulation machinery is located in the intergenic regions of the genome, mostly composed of TEs, consistent with recent findings in other large, complex plant genomes [7,12]”.

32. Comment 32. 237-244. All kind of data are mentioned without a reference to a figure/table.
Response: References to table S5 and figure S10 are added.

33. Comment 33. 257-261. Open chromatin at TSSs and TESs is not epigenetic. It is dependent on the expression level of genes. It has nothing to do with heritable changes to the chromatin structure.
Response (remove): It is not clear what the reviewer meant here, and we believe that we did not make any statements in these two sentences that are incorrect. Active genes are characterized by open chromatin at TSSs and coincide with the epigenetic modifications of chromatin. For example, H3K4me3 epigenetic modification are found in nucleosomes at transcription start sites in humans (Fig. 1 in Ref DOI: 10.1016/j.cell.2007.05.042) or wheat (DOI: 10.1186/s13059-019-1746-8). We tried to modify sentence for clarity: “In the wheat genome, an expression bias towards one of the duplicated gene homoeologs was detected for nearly 27% of gene triplets [30], which also showed epigenetic modifications in promoter or gene coding regions [18,29,31]. Consistent with the patterns of epigenetic modifications, differential MNase digest revealed a high DNS peak immediately upstream of the CDS,….”

34. Comment 34. Figure 2 does not indicate the location of the transcription stop sites.
Response: The figure was corrected. The position on the x-axis is relative to CDS start site. I will add the TSS site with tick on the axis.

35. Comment 35. 305. 19% increase relative to what?
Response: The sentence was modified to remove ambiguity: “…which represents a 19% increase above the average DNS scores for all HC gene models,….”.

36. Comment 36. 316. Reduction relative to what? Region has even lower DNS values than region d.
Response: The sentences were revised to indicate that DNS scores for suppressed homoeologous copies of genes were always compared to DNS scores of non-suppressed copies on other genomes: “Suppression of gene expression in either A or B genomes was accompanied by a significant reduction of DNS scores in regions a, b and d compared to non-suppressed homoeologous gene copies on other genomes (p-values < 10-4; Kruskal-Wallis test). However, for the D genome copies of genes with suppressed expression, a significant reduction in DNS score relative to other homoeologs was observed only in region d (p-values < 0.01; Kruskal Wallis test).”

37. Comment 37. 329. I do not see the data for the 500 bp upstream of the CDS in Figure 4.
Response: The DNS scores for this region shows the same trend (no difference across 5 chromosomal segments) and are now included into revised Fig. 4.

38. Comment 38. 338. A significant DNS score difference compared to what? among which regions?
Response: The text in the manuscript is consistent with the type of statistical test applied (Kruskal Wallis test). The Kruskal Wallis test is a non-parametric version of ANOVA for comparing more than two samples and does not do pair-wise comparisons to say which specific pair of regions is different. Here, the Kruskal Wallis test is used to check whether the analyzed samples (DNS score sets for each of the 5 chromosomal regions) come from the same distribution, and if Kruskal Wallis test’s p-value is below selected threshold (0.05), one can claim that among the compared samples at least one comes from different distribution. We surely can run post-hoc pair-wise comparisons using Mann-Whitney test and then correct for multiple testing to identify statistically significant comparisons among 5 genomic regions. However, comparison of sample means (means of each of the 5 chromosomal regions) showed that the difference from genome-wide average for any of these 5 regions will not be more than 10%. This means that the relative DNS score differences among 5 chromosomal segments for 2 kb regions upstream of genes is not as high as the relative differences observed in the intergenic regions among 5 chromosomal segments. Therefore, finding which of the 5 chromosomal segments is statistically different from others would not add much to what we try to emphasize in this section: genic regions show more or less similar chromatin accessibility across 5 chromosomal segments, whereas intergenic regions’ chromatin accessibility across 5 chromosomal segments vary and show decline from telomere to centromere.

39. Comment 39. 384-385. Cause & effect relations are not shown. So one can only say there is a correlation, not that the position does affect the accessibility.
Response: Corrected. “the relative position of the TE on the chromosome also correlates with the accessibility of their chromatin.”

40. Comment 40. 394-396. There seem to be more with a positive score. e.g. Sakura.
Response: Yes, there are a number of TEs showing positive scores. We highlighted that in the text: “While we observe a highly negative correlation between the incidence of Gypsy superfamily members in a genomic region and chromatin accessibility, we found that the individual Gypsy families demonstrate a wide range of DNS scores…” The ones listed in the text are the three most positive and negative scores for the most common subfamilies. All subfamily score and incidence is listed in Table S9. We have added clarification to the text to indicate that these are the examples of TEs with the most positive scores “… each possess the most positive scores across the TE body in all genomes (Table S9 in Additional File 3; Fig. S14a in Additional File 1)”.

41. Comment 41. 408-410. There is a third with positive scores DTC famc 26.
Response: Thank you for comment. We added this to the text: “Three other CACTA families, Enac (DTC_famc20, DNSall genomes > 0.17), DTC_famc26 (DNSall genomes > 0.17), and Benito (DTC_famc12, DNSall genomes > 0.20) each had positive DNS scores in the TE regions.”.

42. Comment 42. 414. Which Gypsy TEs are overrepresented in gene promoters?
Response: No Gypsy TEs are overrepresented in gene promoters. From Wicker et al. 2018 study in Genome Biology: “Gypsy retrotransposons tend to be excluded from the close vicinity of genes”

43. Comment 43. 413-423. I miss references to tables/figure.
Response: We have added references to Fig. S14 and Table S10.

44. Comment 44. 422-423. You cannot say anything about what is causal. You can only conclde that the average proximity of TEs to genes seems to be positively correlated with their accessibility. This is also true for 441-446. There is a clear correlation, but you do not proof that TEs are the cause of decreased accessiblity of the CDS. Furthermore, this is also true for messages lateron (e.g. 449-450).
Response: We have modified sentence here and further in the text to avoid connecting correlation with causality. “The observed correlation between the proximity of TEs to genes and their sensitivity to the MNase treatment appear to be consistent with the earlier findings showing connection between the spreading of epigenetic modifications near TE insertion sites [9,41]”.

45. Comment 45. 490-498. The text suggests that after showing data in 8e another analysis is done, which you would then expect to end up in another figure panel, but again 8e is being referred to. This is confusing. Then another comparison is done referring to 8f, whereby it is unclear what is different with the previous comparison (different intergenic intervals from distal and centromeric regions).
Response: This paragraph was modified for clarity. Fig. 8e combines two analyses. In the first two boxplots, we show that DNS score is different for two ranges of intergenic intervals sampled across entire genome (8e, first boxplots).The next boxplots show analyses where we tried to remove the effect of centromere-telomere position by analyzing intergenic intervals sampled either from distal or centromeric regions (8e, second and third comparisons). Figure 8f is somewhat redundant and was removed from the revised figure. We retained reference to Table S10 which shows summary of analyses for all intergenic interval ranges.

46. Comment 46. 498-500. Are TEs with lower DNS scores more concentrated close to the centromere? Does this explain the observed lower DNS score the closer to the centromere (even for genes with a distance of <100 kb)?
Response: For TEs, it is difficult to separate the effect of position on chromosome and chromatin accessibility. We do see differences in the concentration of different TEs along the telomere-centromere axis. However, the trends in TE distribution and its chromatin accessibility are not always coinciding with the chromosome-scale trends in chromatin accessibility. For example, Cereba TE shows overall positive mean DNS score, however, its concertation is dramatically increased near centromere.

47. Comment 47. 500-504. It is unclear to me how the data on chromosome 4A can support the finding stated in 498-500. The average distance between genes is very similar, while R1 of 4A is distal, like R1 of 4B and 4D. Still R1 of 4A has lower DNS scores. Maybe R1 of 4A has more TEs that result in lower DNS scores?
Response: This section was modified for clarity as follows: “These results suggest that, in addition to correlation observed between the region’s DNS score and its position on the centromere-telomere axis, there is some connection between the chromatin accessibility and distance between genes. However, the established relationships among these factors are not always straightforward. For example, even though we observed a 2.5-fold reduction in DNS score for the R1 distal region on chromosome 4A (Fig. 1c), in comparison to the same region on chromosome 4B, the average distances between genes in these regions were very similar, with average distances on 4A and 4B being 79.7 kb and 76.4 kb, respectively. This trend on the chromosome 4A-R1 region coincided with 1.5-fold increase in the proportion Gypsy TEs, and 1.5-fold reduction in the proportion CACTA TEs, compared to the R1 regions from other A genome chromosomes, indicative of some connection between chromatin accessibility and the TE composition of intergenic regions.”

48. Comment 48. 536-537. I do not see any CENH3 mapped reads in Fig 9.
Response: Clarified text to say CENH3 centromeric “chromatin localization” instead of mapped reads. CENH3 centromere localization is shown in dark yellow in Fig. 9.

49. Comment 49. Fig 10. Which deciles represent low vs high DNS scores? Where do the SNPs come in? The deciles are for the DNS scores.
Response: This analysis/figure changed based on Reviewer1 comment. We have changed this analysis to now reflect on gene body or gene flanking (within 1kb of gene) SNPs and moved from the deciles to comparing randomly selected SNPs that fall within the most closed and most open regions of the genome. There is a clear pattern that highlights SNPs falling in open regions of the genome explain substantially more variance than for closed regions of the genome for all traits and years phenotyped.

50. Comment 50. 571. What do you mean with higher TE activity? That they amplify more (enlarging the genome)? Something else?
Response: Thank you for comment. The sentence was clarified as follows: “…, which is indicative of more recent TE activity in the D genome lineage compared to that in the A and B genomes”

51. Comment 51. 572. Where do you show the D genome has an increased gene density? On page 20 it is stated that the A genome has an 8 fold increase in gene density at distal regions while the D genome has a 6 fold increase in gene density.
Response: This was inferred based on the smaller genome size and number of genes in the D genome compared to that in the A and B genomes. We have added reference to the wheat genome sequencing paper where this information can be found (10.1126/ science.aar7191).

52. Comment 52. 579. Non-nucleosomal footprint sounds vague.
Response: Modified: “…and occupied by either transcription factors or nucleosomes with conformations making linker DNA more susceptible to digest.”

53. Comment 53. 580. It would be best to add 'proximal' to upstream regulatory regions. 2 kb is still very close by. What about MSFs further upstream than 2 kb?
Response: This section was revised to include discussion of distal cis-regulatory elements that, in the first version of the manuscript, were presented in the results, but were not discussed in detail: “Differential MNase-seq revealed a number of genomic regions detectable only under light digest conditions [3,4], and occupied by either transcription factors or nucleosomes with conformations making linker DNA more accessible. We detected an abundance of MSFs in the proximal regulatory regions, where the levels of chromatin accessibility showed positive correlation with gene expression [1,3,55,56], and were predictive of the unbalanced expression levels among the duplicated homoeologs, supporting the results of chromatin accessibility studies conducted using the DNase-seq and ATAC-seq approaches in wheat [18,29]. However, the majority of MSF (67%) identified in our study were located in the TE-rich intergenic regions, with the average distance of 137 kb from the closest gene, and overlapped with the annotated TEs. These results are consistent with the prevalence of putative distant cis-regulatory elements in crops with large genomes (maize, barley) [7,12,21]. The demonstrated regulatory potential of TE elements derived from regions with open chromatin [7,22] suggests that genome size expansion driven by TE proliferation in the wheat genomes have a great potential to diversify gene expression regulatory pathways. However, TE space expansion does not directly correlate with MSF frequency, which appear to be conditioned by the regional gene density. The enrichment of MSF in the distal chromosomal regions with high gene/low TE density compared to MSF frequency in the pericentromeric regions characterized by low gene/high TE density is consistent with this hypothesis, and supported by a finding that total size of distal accessible chromatin regions does not scale linearly with increase in genome size [12].”

54. Comment 54. 587. Bias in gene expression levels?
Response: Changed to “unbalanced gene expression level”.

55. Comment 55. 586-589. This is not just true for wheat, also for other organisms, including animals.
Response: The reference to humans and a review covering regulatory epigenome studies is added.

56. Comment 56. 590-591. You observed a correlation.
Response: Modified.

57. Comment 57. 598. The type of TEs is not addressed in the sentences before. So better use 'our' results in 596.
Response: This paragraph is modified as suggested.

58. Comment 58. 616-619. Your data in figure 1c indicated there is some change towards more open chromatin.
Response: Yes, we do see some changes, but these changes are not statistically significant, and also likely caused by the presence of short distal segment from the ancestral chromosome 4A in modern 4A (aR1 segment in Fig. 1d). This sentence was modified: “The lack of substantial changes in chromatin since the occurrence of chromosome 4A’s structural re-arrangement suggests that global chromatin states tend to remain stable, at least within short evolutionary time scales,….”.

59. Comment 59. 633. TE silencing is most effective when TEs are retained in the genome: this sentence is confusing. Compared to what? IS there another option than being in the genome?
Response: We apologize for this confusing statement. Here we wanted to describe conclusions based on the modeling of TE propagation, response of a host genome to TE propagation, and accumulation of silenced TEs (Roessler et al., Genome Biol Evol. 2018;10:803–15). Retained copies of TEs could be the major source of siRNAs that can mediate the silencing of newly propagated TE copies through RNA-directed DNA methylation pathway. Thereby, the retention of duplicated copies of TEs in genome could lead to more effective silencing on newly duplicated TEs providing a mechanism for a host genome to control TE activity. In turn, these processes could lead to accumulation of TEs in genome and increase in genome size. The sentence was modified as follows: “These results are in line with the predictions based on the modeling of TE propagation, response of a host genome to TE propagation, and accumulation of silenced TEs in a host genome [60]. This model suggests that lower TE deletion rates resulting in TE accumulation could lead to more effective silencing of the retained copies of TEs through siRNA-mediated DNA methylation pathway and increase in genome size [60].”

60. Comment 60. 634-638. The messages here are also not entirely clear. For example what is meant with removal? Removal through which process? Recombination? A cut mechanism without being followed by the 'paste' part of the process? why would recent TE bursts be accompanied by less effective removal of TEs from low recombining regions?
Response: When we talk about TE removal here, we mean TE allele (by allele we mean specific location in the genome) loss from population and fixation of alleles without TE insertion. The origin of “TE loss” alleles can be associated with physical deletion of TE element due to recombination between LTRs (Class 1 TEs, most abundant in the large plant genomes), non-homologous recombination, un-equal crossing over, etc.

We do agree that this section need to be more clearly written, and it has been modified as follows: “However, this model does not explain the origin of gene density gradient along the telomere-centromere axis and preferential accumulation of TEs in the pericentromeric regions. If TE insertion near genes is negatively selected due to its detrimental effects on gene expression [59], and at the same time TE retention is under positive selection as a part of pathways needed to control TE proliferation [60], one might suggest that TE distribution is defined by the efficiency of selection in different parts of a genome, which in turn is strongly influenced by recombination rate. Strong suppression of recombination in the pericentromeric regions of large wheat chromosomes, and associated with it reduction in the efficiency of selection [61,62] could potentially create conditions for the disproportional accumulation of TEs in the pericentromeric regions compared to the distal regions”.

61. Comment 61. 642-643: also true for ref 49, 51 (and others)
Response: Additional references added.

62. Comment 62. 652-655. Figure 1 and 9 indicates the highest scores are observed at the chromosome ends.
Response: The sentence was revised. We did see on few chromosomes the highest DNS peaks, but overall centromeric DNS scores are still lower that the DNS scores of the telomeric regions.

63. Comment 63. 663-666. Can you explain why the DNS scores and Cereba LTR density would be affected by an artifact of genome assembly and the immunoprecipiation signal not?
Response: The sentence was revised as follows: “…CENH3 signal detected using CENH3 immunofluorescence [46] and immunoprecipitation [35,44], suggesting that not in all cases coincidence of DNS score and Cereba LTR density peaks is predictive of centromere location.”.

64. Comment 64. 676. See als Ricci..Schmitz..Zhang et al and Lu … schmitz et al 2019 (Nature plants).
Response: We should note that we did not have chance to include these references because they have been published several months after we have submitted our manuscript to Genome Biology. The references are added.

65. Comment 65. 679. In Oka et al DNase I-seq data predict enhancers genome wide, not only for the genes indicated.
Response: The sentence was modified and new references added as follows: “…was also previously demonstrated for maize where DNase I chromatin accessibility was used to predict distantly located enhancers genome-wide and for the b1, bx1 and tb1 genes [7,12,21].”

**Second round of review**

**Reviewer 1**

Thank you for revising the manuscript to address my concerns. I also think that the shortening of some sections has improved the manuscript for readability.
